# Study of Oxidation and Polarization-Dependent Optical Properties of Environmentally Stable Layered GaTe Using a Novel Passivation Approach

**DOI:** 10.3390/nano9111510

**Published:** 2019-10-23

**Authors:** Mounika Kotha, Thomas Murray, David Tuschel, Spyros Gallis

**Affiliations:** 1 College of Nanoscale Science and Engineering (CNSE), SUNY Polytechnic Institute (SUNY Poly), Albany, NY 12203, USA; mkotha@sunypoly.edu (M.K.); tmurray@sunypoly.edu (T.M.); 2 HORIBA Scientific, Piscataway, NJ 08854, USA; david.tuschel@horiba.com

**Keywords:** gallium telluride, 2D materials, hydrogen silsesquioxane, anisotropy, Raman spectroscopy, photoluminescence, pseudo-one-dimensional materials

## Abstract

Emerging two-dimensional gallium chalcogenides, such as gallium telluride (GaTe), are considered promising layered semiconductors that can serve as vital building blocks towards the implementation of nanodevices in the fields of nanoelectronics, optoelectronics, and quantum photonics. However, oxidation-induced electronic, structural, and optical changes observed in ambient-exposed gallium chalcogenides need to be further investigated and addressed. Herein, we report on the thickness-dependent effect of air exposure on the Raman and photoluminescence (PL) properties of GaTe flakes, with thicknesses spanning in the range of a few layers to 100 nm. We have developed a novel chemical passivation that results in complete encapsulation of the as-exfoliated GaTe flakes in ultrathin hydrogen–silsesquioxane (HSQ) film. A combination of correlation and comparison of Raman and PL studies reveal that the HSQ-capped GaTe flakes are effectively protected from oxidation in air ambient over the studied-period of one year, and thus, preserving their structural and optical characteristics. This contrasts with the behavior of uncapped GaTe, where we observe a significant reduction of the GaTe-related PL (~100×) and Raman (~4×) peak intensities for the few-layered flakes over a period of few days. The time-evolution of the Raman spectra in uncapped GaTe is accompanied by the appearance of two new prominent broad peaks at ~130 cm^−1^ and ~146 cm^−1^, which are attributed to the formation of polycrystalline tellurium, due to oxidation of ambient-exposed GaTe. Furthermore, and by leveraging this novel passivation, we were able to explore the optical anisotropy of HSQ-capped GaTe flakes. This is caused by the one-dimensional-like nature of the GaTe layer, as the layer comprises Ga–Ga chains extending along the *b*-axis direction. In concurrence with high-resolution transmission electron microscopy analysis, polarization-dependent PL spectroscopy was used to identify the *b*-axis crystal direction in HSQ-capped GaTe flakes with various thicknesses over a range of wavelengths (458 nm–633 nm). Thus, our novel surface-passivation offers a new approach to explore and reveal the physical properties of the layered GaTe, with the potential of fabricating reliable polarization-dependent nanophotonics with structural and optical stability.

## 1. Introduction

A recent surge of interest in two-dimensional (2D) layered semiconductors [1], such as metal chalcogenides (MCs), is attributed to their remarkable properties, which established them as a distinct class of materials with the potential for immense technological applications in the fields of electronics [2], optoelectronics [3,4], sensing [5,6] and quantum information technology [7,8]. Monolayer MCs, such as molybdenum and tungsten chalcogenides, have shown strong photon emission, owing to their transformation from indirect- to direct-gap semiconductors [9], but conversely gallium chalcogenides, such as gallium telluride (GaTe) and gallium selenide (GaSe), possess a direct-gap for a range of nanoscale thicknesses, thus, relaxing strict synthesis requirements associated with atomically-thin semiconductors [10,11].

**Figure 1:**
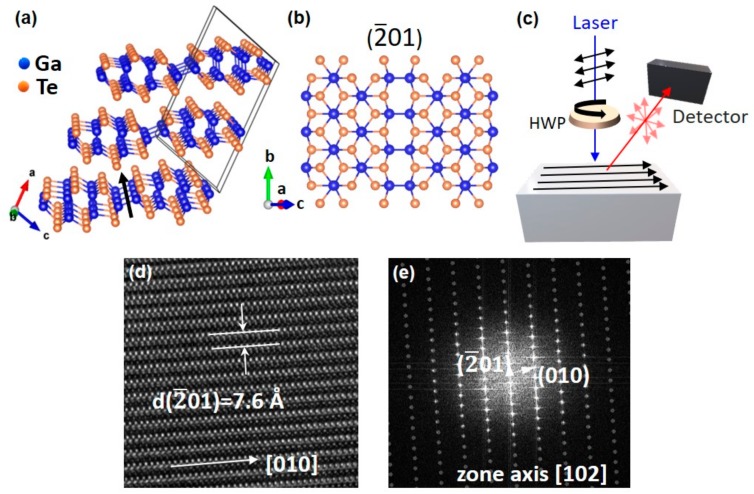
Crystal structure of layered gallium telluride (GaTe) flakes; (**a**) Schematic showing the monoclinic unit cell and the *b*-axis (black arrow) of the crystal; (**b**) Projection along normal to ($\bar{2}$01) of monolayer GaTe showing the formation of pseudo-1D chains of Ga–Ga bonds (blue spheres) along the *b*-axis; (**c**) Schematic of the experimental setup for polarization-dependent photoluminescence measurements using a half waveplate (HWP); (**d**) High-resolution transmission electron microscopy (HRTEM) image of a GaTe flake showing the interlayer spacing for ($\bar{2}$01) and [010] chain, *b*-axis, direction. The spacing between the lattice fringes was ~7.6 Å, which is close to the reported thickness (~7.5 Å) for monolayer GaTe [14]; (**e**) FFT (Fast Fourier Transform) image along the [102] zone axis showing periodicity of {$\bar{2}$01} and {010} family of planes.

Studies on the physical behaviors of layered GaTe are still at the early stages of fundamental research, and thus, are of significant interest from a scientific and technological point of view. Quasi-2D GaTe is a layered, direct-bandgap semiconductor having a room-temperature bandgap of approximately 1.66 eV [10,11]. GaTe is typically a p-type material, with each monolayer comprising a Te-Ga-Ga-Te assembly (Figure 1), adding to the set of candidate layered semiconductors for the fabrication of van der Waals heterostructures and devices [10,12,13]. Recent reports have opened a whole new world of exciting opportunities for the use of mechanically-exfoliated and CVD-grown GaTe in van der Waals heterostructure nanoelectronics [13] in nonlinear optics applications [14], in radiation detectors [15], and in optoelectronics, such as in photodetectors, due to the high photoresponsivity (10^4^ A/W) and short response time (6 ms) demonstrated in layered GaTe [16,17].

Unlike other gallium chalcogenides, GaTe has two-thirds of the Ga–Ga bonds perpendicular to the layer and one-third almost in the plane of the layer. These in-plane Ga–Ga bonds form pseudo-1D chains along the *b*-axis, [010] crystal direction in the ($\bar{2}$01) 2D plane (Figure 1b). This structural anisotropy in monoclinic GaTe leads to direction-dependent light-matter interactions [18,19], resulting in strong anisotropic optical and electrical responses crucial for applications in polarization-sensitive devices, such as polarized detectors, sensors, anisotropic memory, and quantum key distribution for quantum cryptography. Furthermore, highly anisotropic GaTe has been reported to exhibit a defect-related, below-bandgap bright photoluminescence (PL) emission [20].

However, an important obstacle in the practical implementation of 2D and quasi-2D gallium chalcogenides for electronic and photonic applications is their surface instability under ambient conditions. For example, unpassivated surfaces of single and multi-layered GaTe have been reported to exhibit low ambient stability (e.g., air oxidation) leading to significant conduction band restructuring [11], an anisotropic to isotropic structural transition [21], and low PL emission yield caused by carrier dissociation via surface states [10].

In this work, we studied the effect of ambient and oxygen exposure on the structural and optical properties of mechanically exfoliated GaTe flakes as a function of time and flake thickness. A systematic set of experiments were designed to study the time-evolution behavior of Raman and PL properties of flakes with thicknesses from a few layers to 100 nm. Furthermore, we developed a novel surface passivation where the as-exfoliated GaTe flakes are encapsulated in ultrathin hydrogen-silsesquioxane (HSQ) film, thus effectively protecting the flakes against ambient oxidation, and thus, maintaining their pristine structural and optical characteristics. Moreover, annealing, Auger electron spectroscopy (AES), and X-ray photoelectron spectroscopy (XPS) studies provide further insight into the oxidation process of GaTe.

By leveraging this novel passivation, we were able to explore the anisotropy in the optical properties of HSQ-capped GaTe flakes. Polarization-dependent PL spectroscopy (schematically shown in Figure 1c) was used to identify the *b*-axis crystal direction in HSQ-capped GaTe flakes with various thicknesses over a range of wavelengths (458 nm–633 nm). We observed a high PL intensity when the electric field of the excitation laser light was parallel to the *b*-axis of HSQ-capped GaTe. The explanation for such an observation is discussed in detail in the later sections of this article. Furthermore, the *b*-axis, [010] crystal direction, identified through polarization-dependent PL was also confirmed using high-resolution transmission electron microscopy (HRTEM) and selected area electron diffraction (SAED) patterns taken along the [102] zone axis (Figure 1d,e). Raman spectroscopy was also explored for the identification of the *b*-axis, but the results revealed a dependence on both the thickness and excitation laser wavelength.

## 2. Materials and Methods

The GaTe flakes were obtained by mechanical exfoliation of Bridgman-grown bulk GaTe crystals. For the passivation study experiments, we spin-coated the as-exfoliated flakes with 40 nm HSQ (XR-1541, Dow Corning, MI, USA) at 4000 rpm for 60 s. The samples then underwent a thermal process at 300 °C for 30 min under Argon ambient at atmospheric pressure. Steady-state room-temperature PL and Raman measurements were carried out with a LabRAM HR-800 (HORIBA Scientific, Piscataway, NJ, USA) system utilizing 532 and 633 nm excitation lines with the use of optical density filters, to avoid laser-induced sample degradation [22]. Furthermore, a home-built micro-PL system composed of an argon laser (Beamlok 2065-7S, Spectra-Physics, Santa Clara, CA, USA), a microscope cryostat, a 50× objective lens, a scanning nano-stage (1 nm resolution) coupled to an FLSP920 spectrometer (Edinburgh Instruments, Livingston, UK) were utilized for PL and time-resolved PL (TRPL) measurements. The fluorescence lifetimes were extracted from the measured PL decay transients after reconvolution analysis to account for the instrument response function (IRF), in accordance with our previously reported analysis [23]. Flake thickness was identified using atomic force microscopy (AFM) with a Dimension Icon system (Bruker, Billerica, MA, USA). High-resolution AES with a depth resolution of ~30 Angstroms (Phi-680, Physical Electronics, MN, USA) and XPS (ThetaProbe, Thermo Fisher Scientific, MA, USA) were employed to provide elemental and chemical state information. The polarization-dependent Raman/PL studies at 532 nm and 633 nm were conducted by shining linearly polarized light on a sample placed on a rotating stage. The polarization-dependent photoluminescence studies at 458 nm and 476 nm were conducted using a half waveplate to rotate the polarization of the excitation laser (Figure 1c). TEM analysis was conducted on a Titan 80-300 (FEI Company, OR, USA) operated at 300 kV in conventional TEM mode. Diffraction was performed using a 40 µm selected area aperture.

## 3. Results and Discussion

We monitored and recorded PL and Raman spectra of exfoliated monoclinic GaTe flakes beginning from freshly cleaved to year-old GaTe flakes kept in ambient air. The point in time at which Raman and PL spectra of the as-exfoliated samples were measured is denoted as the “0 h” point and considered as the reference time for the samples.

### 3.1. Raman Spectroscopy

**Figure 2:**
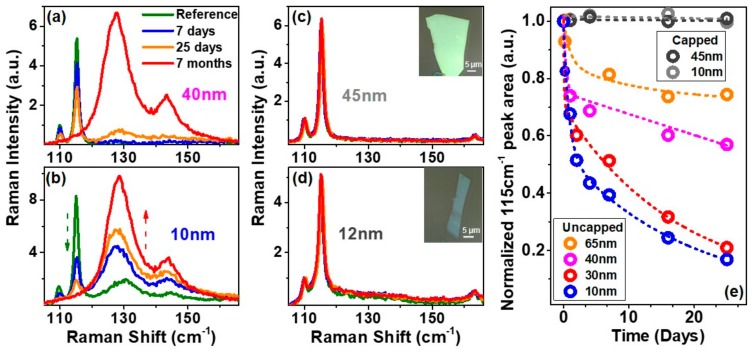
Room temperature Raman spectra of mechanically exfoliated GaTe flakes with a thickness of (**a**) 40 nm and (**b**) 10 nm at the reference point for uncapped flakes and after exposure to ambient air for 7 days, 25 days, and 7 months; Raman spectra of HSQ-capped flakes with thickness (**c**) 45 nm (optical image shown in inset) and (**d**) 12 nm (optical image shown in inset) at the reference point for HSQ-capped flakes and after exposure to ambient air for 7 days, 25 days, and 7 months; (**e**) The normalized 115 cm^−1^ Raman peak area for uncapped flakes of thicknesses 65 nm, 40 nm, 30 nm, 10 nm, and HSQ-capped flakes representative of the thin (8 nm–15 nm) and thick (30 nm–60 nm) regimes are shown in light grey and dark grey dotted lines, respectively.

Figure 2a,b present the time-evolution of the uncapped GaTe Raman spectra with thicknesses 40 nm and 10 nm, respectively. These are representative thicknesses of the two thickness-regimes investigated in this work: a thick regime with flake thickness range between 30 and 60 nm, and a thin regime with thickness range between 8 and 15 nm. We observed that the intensity of the A_g_-Raman peak around 115 cm^−1^ for the 10 nm flake is higher as compared with the 40 nm flake, which can be explained considering optical interference effects of the stacked GaTe/SiO_2_/Si materials [24]. In addition to the two expected monoclinic GaTe-induced Raman peaks at 109 and 115 cm^−1^, two broad peaks at 130 and 146 cm^−1^ begin to emerge after 48 h of air exposure in the case of the thick-regime samples (Figure 2a). Conversely, for samples in the thin regime, these new peaks are seen even at the reference time (Figure 2b). After 7 months, we see a complete disappearance of the monoclinic GaTe-induced Raman peaks, and the spectra are dominated with two broad peaks ~130 cm^−1^ and 146 cm^−1^, which can be attributed to polycrystalline (poly) Te. These poly-Te Raman peaks (~130 cm^−1^ A_1_ Raman and ~146 cm^−1^ E Raman modes) have also been observed in several tellurium-based compounds [25,26,27,28].

To further deduce that the above changes were caused by the exposure of GaTe flakes to air, corresponding HSQ-capped GaTe samples were also investigated. To this end, we did not observe any change in the Raman spectra for the HSQ-caped samples, even after 7 months of exposure to air (Figure 2c,d). The 115 cm^−1^ peak from spectra collected on different days were fitted with a Lorentzian function. We then normalized the fitted 115 cm^−1^ peak areas to the peak area of the spectrum at the reference time, and the resulting data were plotted as a function of time for both the thick- and thin-regime samples (Figure 2e).

The normalized A_g_ Raman mode peak areas of the uncapped GaTe samples decreased with time. The time rate decrease in the GaTe Raman signal was much faster for the thin flakes. For example, for the 10 nm sample, there was a drop of 84% after 25 days in contrast to the 65 nm sample, which dropped by 26% within the same time frame. The decrease rate was considerably higher within five hours upon exfoliation irrespective of the thickness followed by a slower rate, which is thickness dependent (exponential decrease for the thin flakes) until complete diminishing of peaks after 7 months.

### 3.2. Photoluminescence Spectroscopy

**Figure 3:**
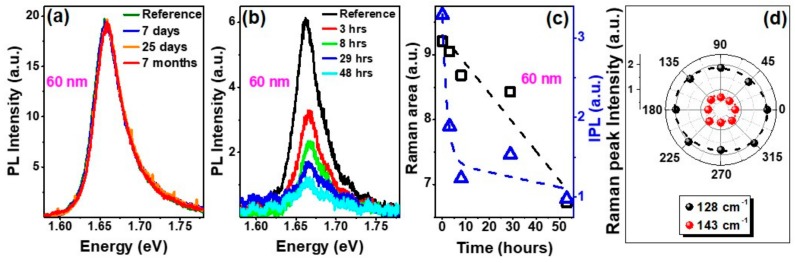
Room temperature photoluminescence (PL) spectra of mechanically exfoliated (**a**) HSQ-capped and (**b**) uncapped 60 nm-thick GaTe flakes; (**c**) 115 cm^−1^ Raman peak area (black) and integrated PL peak area (blue) plotted with respect to time for the uncapped 60 nm sample shown in (b); (**d**) Incident laser polarization dependence of the Raman peak intensity at 128 cm^−1^ and 143 cm^−1^.

As mentioned in the introduction, preserving the PL properties of the exfoliated flakes is imperative for potential novel photonic and optoelectronic devices based on these materials. Figure 3a shows the time-evolution of the PL spectra for a 60 nm HSQ-capped GaTe flake at the reference time, after 7 days, after 25 days, and after 7 months of ambient air exposure. The PL behavior in HSQ-capped GaTe remained unchanged even after prolonged 7-month air exposure in contrast to the behavior of its uncapped counterpart, where the PL intensity substantially decreased within hours of exposure (Figure 3b). We observed that in the case of the uncapped samples, the integrated PL dropped by 70% within 48 h upon exfoliation. In Figure 3c, we present the integrated PL intensity and 115 cm^−1^ Raman peak area for the 60 nm uncapped flake as a function of time. Unlike the linear trend in the Raman peak area, the integrated PL intensity decreased substantially in a non-linear fashion within the same time interval, further highlighting the importance for effective passivation to preserve the optical properties of layered GaTe.

### 3.3. Auger and XPS Spectroscopy

**Figure 4:**
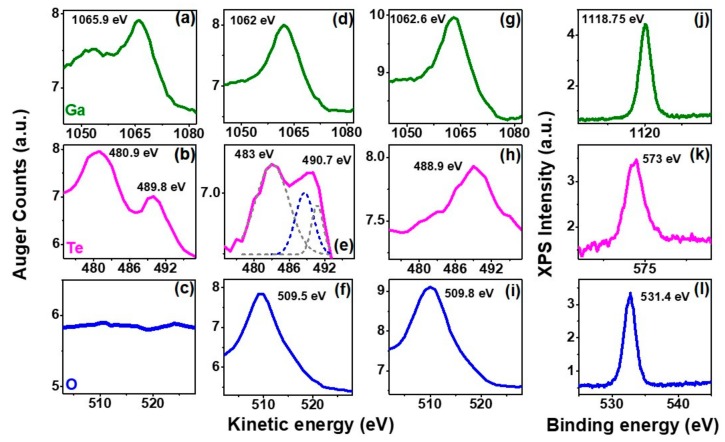
Auger spectra of Ga, Te, and O, respectively, (**a**–**c**) for a 55 nm HSQ-capped GaTe flake exposed to ambient air for 43 days; (**d**–**f**) for a 68 nm uncapped GaTe flake exposed to ambient air for 20 days; (**g**–**i**) for a 190 nm flake exposed to ambient for 5 months and subsequently annealed at 300 °C for 30 min in argon ambient. The deconvoluted peak at ~488 eV (blue) shown in (e) belongs to the KL_1_L_23_ Auger transition for oxygen bonded to gallium in GaO_x_. XPS spectra of Ga, Te, and O, respectively, (**j**–**l**) for an uncapped ambient-exposed GaTe flake.

Figure 4 shows the undifferentiated Auger spectra for gallium (Ga), tellurium (Te), and oxygen (O). For the ambient-exposed uncapped flake, we observed a Ga L_3_M_23_M_23_ kinetic energy (~1062 eV) such as that of Ga in GaO_x_ (Figure 4d) [29]. An apparent ~4 eV energy redshift for the uncapped flake was seen with respect to the expected kinetic energy [30] for Ga in GaTe (~1066 eV) in HSQ-capped GaTe (Figure 4a), and the appearance of the ~488 eV Auger peak which corresponding to the O KL_1_L_23_ in GaO_x_ (deconvoluted blue dash-line in Figure 4e) [31], suggests that Ga in uncapped GaTe flakes transformed to GaO_x_ over exposure to ambient air. Moreover, the Te M_4_N_45_N_45_ peaks (~483 eV and ~491 eV peaks eV) in the uncapped flake (deconvoluted grey dash-lines in Figure 4e) can be attributed to the formation of Te [29]. The absence of oxygen signal in the HSQ-capped flake (Figure 4c), as compared to the KL_23_L_23_ peak [32] of oxygen observed ~509 eV in the uncapped flake (Figure 4f), is an additional manifestation that the HSQ-capped GaTe samples were effectively protected from oxidation in air ambient over the studied-period. XPS of the uncapped ambient-exposed GaTe flakes showed the appearance of Ga 2p_3/2_ (Figure 4j) and Te 3d_5/2_ (Figure 4k) peaks at binding energies of ~1118.8 eV and ~573 eV, respectively, corresponding to the formation of GaO_x_ [33,34] and Te [35]. Furthermore, we did not observe any oxidation/degradation in GaTe flakes after annealing at 200 °C in pure oxygen ambient (Figure S1), which is consistent with previous studies where water vapor or oxygen alone did not support the oxidation process [11,21].

This oxidation behavior has been reported to be associated with the formation of an oxygen-chemisorbed phase in GaTe [11] and/or the formation of tellurium oxide/dioxide (TeO_x_/TeO_2_) [21]. However, our combinational XPS, Auger, and Raman study suggests the formation of poly-Te and gallium oxide (GaO_x_), which have been previously reported for other gallium chalcogenide-based compounds [36,37]. The redshift of the Te Raman modes (~130 cm^−1^ and 146 cm^−1^) with an increase in ambient exposure time (Figure 2b) is consistent with Raman observations where the thickness of Te increases [38]. The polarization-dependent Raman spectra of these peaks show an isotropic dependence (Figure 3d) and can be attributed to the polycrystalline nature of the formed tellurium.

Additionally, we found interesting results when we annealed the oxidized GaTe flakes at 300 °C for 30 min in argon ambient. The annealing outdiffused all the tellurium (absence of 483 eV and 491 eV peaks) in the flakes leading to the formation of GaO_x_, as observed in the Auger study (Figure 4g–i). The absence of Raman 130 cm^−1^ or 146 cm^−1^ peaks from these flakes further confirms our experimental observations of the formation of poly-Te in oxidized GaTe (Figure S2). Unlike TeO_2_, which can only be outdiffused at higher temperatures (~725 °C) in GaTe, Te was evaporated due to high volatility at low temperatures [39].

### 3.4. Time-Resolved Photoluminescence (TRPL) Spectroscopy

**Figure 5:**
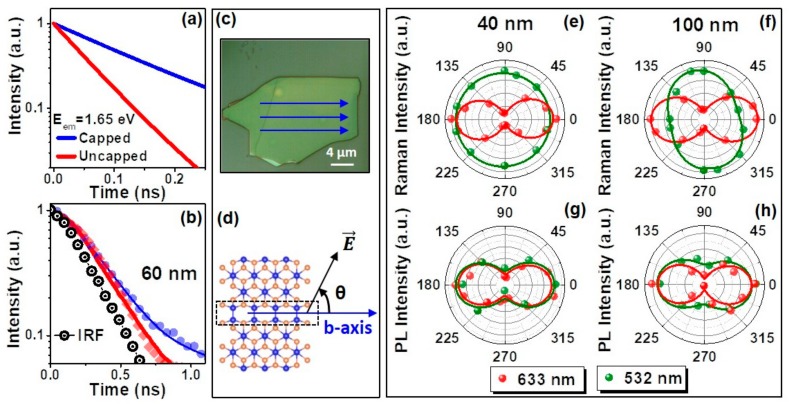
(**a**) Representative reconvoluted room-temperature PL decay transients at 1.66 eV emission, and (**b**) measured PL decay transients of a capped (blue) and as-exfoliated uncapped GaTe flake (red) acquired using a pulsed diode laser source [λ = 405 nm (3.06 eV), ~50 ps full width at half maximum (FWHM)]. The measured PL decays were fitted using reconvolution analysis based on the instrument response function (IRF) (solid lines) [23]. (**c**) Optical image of a 100 nm-thick GaTe flake shown along with the *b*-axis (blue lines) identified using TEM; (**d**) Schematic showing the angle (*θ*) between the laser polarization direction and the crystal *b*-axis used for polarization-dependence measurements. (**e**,**f**) Polarization dependence Raman peak (115 cm^−1^) intensity and (**g**,**h**) PL peak (1.66 eV) intensity polar plots of 40 nm and 100 nm thick HSQ-capped flakes, respectively, for the excitation wavelengths of 633 nm (red) and 532 nm (green). A high PL intensity was observed when the electric field of the excitation laser light was parallel to the *b*-axis of HSQ-capped GaTe (*θ* = 0°), in agreement with TEM analysis. The equation, $y=y_{0}+A\left(cos^{2}\theta \right)$ was used for fitting the polar plots.

In the case of capped-GaTe flakes, surface states which can interact with air ambient, are passivated as the HSQ encapsulation acts as a protective layer over the underlying GaTe. Therefore, this can substantially inhibit non-radiative recombination associated with surface states leading to an increase in radiative recombination processes and hence, the PL emission observed in capped-GaTe (Figure 3). This is further reflected in TRPL measurements at 1.66 eV emission, which revealed a shorter PL decay lifetime τ of ~60 ± 4 ps for a representative as-exfoliated GaTe flake measured in nitrogen ambient, and a longer τ of ~140 ± 25 ps in the HSQ-capped GaTe flake (Figure 5a,b).

### 3.5. Polarization-Dependent Raman and Photoluminescence Spectroscopy

Our effective encapsulation process using HSQ provides a platform for studying the anisotropic properties of pristine GaTe flakes through polarization-dependent Raman and PL spectroscopy. Our study shows that the polarization dependence of the Raman mode at 115 cm^−1^ highly depended on excitation wavelength and flake thickness (Figure 5e,f), thus making it more complicated to determine the *b*-axis direction of GaTe using Raman spectroscopy. For example, there was a complete extinction of the polarization anisotropy at 532 nm as compared with 633 nm for a 40 nm flake. While a 100 nm flake showed anisotropic dependence at both wavelengths, there was a shift in the anisotropy axis. Normally, the Raman polarization response would be dictated by the crystal class to which the material belongs and the symmetry species of the particular phonon; neither depend upon laser excitation wavelength or sample thickness. However, as Huang and coworkers showed in their study of layered GaTe, phonon coupling to electronic transitions occur that depend upon the thickness of the GaTe [18]. Consequently, resonance Raman effects account for the dependence of the Raman polarization response on both excitation wavelength and GaTe thickness.

Conversely, we observed similar dependences of the GaTe room-temperature PL peak emission (1.66 eV) anisotropy using several excitation wavelengths, such as 633 nm, 532 nm, 476 nm, and 458 nm and various thicknesses (Figure 5g,h and Figure S3). This anisotropy is different from that observed in black phosphorus, which is independent of the exciting polarization direction [40]. The origin of the observed PL polarization anisotropy can be understood with classical electromagnetic theory [41], where the in-plane Ga–Ga bonds running along the *b*-axis of the GaTe monoclinic crystal structure behave as pseudo-one-dimensional (1D) chains/nanowires (Figure 1a,b). Due to this pseudo-1D nature of the chains along the *b*-axis and their effective permittivity, ε(ω), contrast with the surrounding medium [42], when the electric field of the polarized laser excitation is parallel to the 1D chains, it induces a weak depolarization field in the chains (the wavelength of the excitation laser light is much smaller than the chain length). Therefore, the electric field amplitude inside the chains is not attenuated, and the optical absorption is strong. However, when the field (*E_out_*) is perpendicular to the chain direction, a strong depolarization field is induced in the 1D chains, and thus the electric field amplitude inside the chains (*E_in_*) is attenuated yielding much weaker absorption. Considering a cylindrical nanowire geometry, the electric field amplitude is attenuated as follows [43]:

$$E_{in}=\frac{2}{1+\varepsilon \left(\omega \right)}E_{out},$$

while the polarization anisotropy contrast (*ρ*) can be quantified using the equation

$$\rho =\frac{I_{∥}-I_{⊥}}{I_{∥}+I_{⊥}},$$

where $I_{∥}$ and $I_{⊥}$ are the integrated PL intensity along a direction parallel and perpendicular to the *b*-axis, respectively. For example, we observed ρ of ~0.6 for the 40 nm flake at 532 nm, which is comparable to other pseudo-1D materials such as ZrS_3_ [44]. Thus, PL spectroscopy, in agreement with our TEM results, can be employed in identifying the *b*-axis in such anisotropic material systems. This is imperative towards advancements in the fabrication of polarization-dependent devices based on the generation and detection of polarized light, such as polarized photodetectors and light sources, using environmental-stable GaTe flakes.

## 4. Conclusions

In conclusion, Raman, PL, and chemical studies revealed that the HSQ-capped GaTe flakes were effectively protected from oxidation, whereas in the case of uncapped GaTe, we observed a significant reduction of the GaTe-related PL and Raman peak intensities. We suggest that there were oxidation-induced changes in the ambient-exposed uncapped GaTe, leading to the formation of GaO_x_ and polycrystalline Te evidenced through a combinational and systematic study which has not been previously reported for layered GaTe. Our novel surface-passivation has a dual role. Together with effectively passivating and protecting the flakes, it offers the capability to simplify the integration process of fabricating GaTe-based nanodevices, given HSQ is the most commonly used resist for electron beam lithography. Moreover, polarization-dependent PL spectroscopy was used to identify the *b*-axis crystal direction in HSQ-capped GaTe flakes with various thicknesses. Our holistic approach can thus further help explore the physical properties of layered GaTe, towards the practical realization of polarization-dependent nanophotonic devices with structural, and photoluminescence stability.

## Supplementary Materials

The following are available online at https://www.mdpi.com/2079-4991/9/11/1510/s1, Figure S1: Effect of annealing on as-exfoliated GaTe flakes in a pure oxygen ambient at 200 °C for 30 minutes; Figure S2: Effect of annealing on oxidized flakes in an argon ambient at 300 °C for 30 minutes; Figure S3: Polarization dependence of PL peak intensity (1.66 eV) of a 100 nm GaTe flake measured using excitation wavelength of (a) 458 nm; (b) 476 nm.
