# Supplementary material for: Study of Oxidation and Polarization-Dependent Optical Properties of Environmentally Stable Layered GaTe Using a Novel Passivation Approach
Source: Nanomaterials (Basel). 2019 Oct 23;9(11):1510. doi: 10.3390/nano9111510 (PMC6915591; doi:10.3390/nano9111510)
Supplement: Supplementary file 1 [file nanomaterials-09-01510-s001.pdf]

Supplementary material

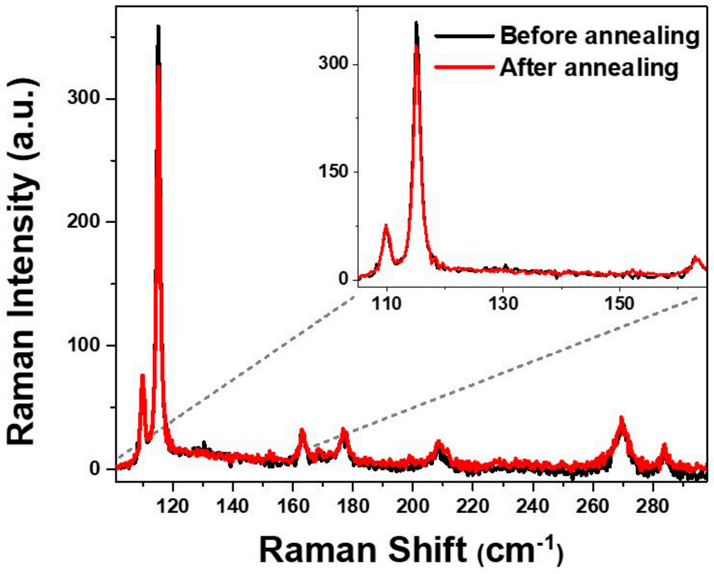

Figure S1: Effect of annealing on as-exfoliated GaTe flakes in a pure oxygen ambient at 200 °C for 30 minutes

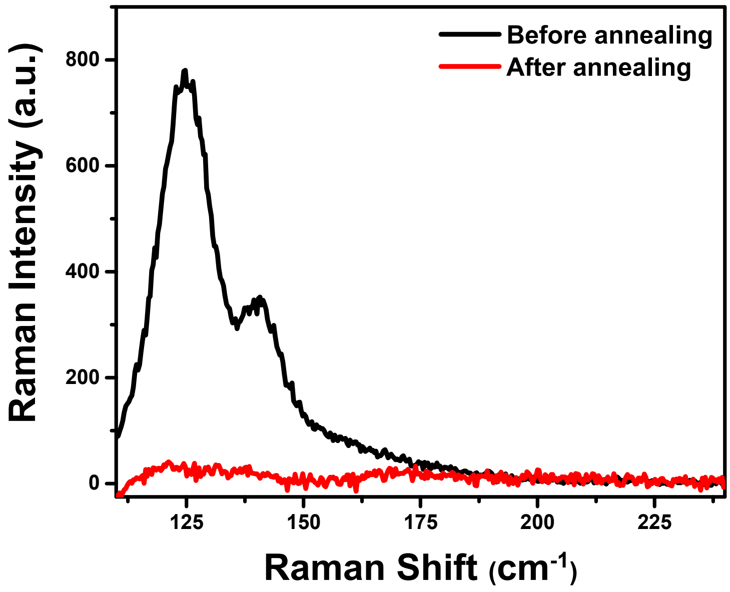

Figure S2: Effect of annealing on oxidized flakes in an argon ambient at 300 °C for 30 minutes

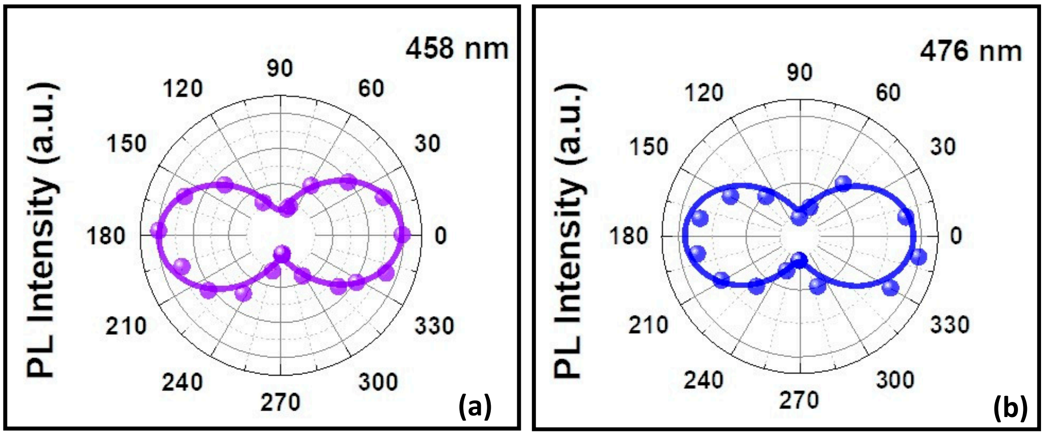

Figure S3: Polarization dependence of PL peak intensity (1.66 eV) of a 100 nm GaTe flake measured using excitation wavelength of (**a**) 458 nm; (**b**) 476 nm
